# The intestinal barrier as an emerging target in the toxicological assessment of mycotoxins

**DOI:** 10.1007/s00204-016-1794-8

**Published:** 2016-07-14

**Authors:** Peyman Akbari, Saskia Braber, Soheil Varasteh, Arash Alizadeh, Johan Garssen, Johanna Fink-Gremmels

**Affiliations:** 1 Division of Veterinary Pharmacology, Pharmacotherapy and Toxicology, Institute for Risk Assessment Sciences, Utrecht University, Yalelaan 104, 3584 CM Utrecht, The Netherlands; 2 Division of Pharmacology, Utrecht Institute for Pharmaceutical Sciences, Faculty of Science, Utrecht University, 3584 CG Utrecht, The Netherlands; 3 Nutricia Research, 3584 CT Utrecht, The Netherlands

**Keywords:** Mycotoxins, Tight junction proteins, Intestinal permeability, Mucosal inflammation

## Abstract

Mycotoxins, the secondary metabolites of fungal species, are the most frequently occurring natural food contaminants in human and animal diets. Risk assessment of mycotoxins focused as yet on their mutagenic, genotoxic and potential carcinogenic effects. Recently, there is an increasing awareness of the adverse effects of various mycotoxins on vulnerable structures in the intestines. In particular, an impairment of the barrier function of the epithelial lining cells and the sealing tight junction proteins has been noted, as this could result in an increased translocation of luminal antigens and pathogens and an excessive activation of the immune system. The current review aims to provide a summary of the available evidence regarding direct effects of various mycotoxins on the intestinal epithelial barrier. Available data, based on different cellular and animal studies, show that food-associated exposure to certain mycotoxins, especially trichothecenes and patulin, affects the intestinal barrier integrity and can result in an increased translocation of harmful stressors. It is therefore hypothesized that human exposure to certain mycotoxins, particularly deoxynivalenol, as the major trichothecene, may play an important role in etiology of various chronic intestinal inflammatory diseases, such as inflammatory bowel disease, and in the prevalence of food allergies, particularly in children.

**Electronic supplementary material:**

The online version of this article (doi:10.1007/s00204-016-1794-8) contains supplementary material, which is available to authorized users.

## Introduction

Since the early discovery of aflatoxins as food and feed contaminants, risk assessment of mycotoxin exposure has been initially focused on their mutagenic, genotoxic and potentially carcinogenic effects, as major human health risks (Bennett and Klich 2003; Liu and Wu 2010; Wu et al. 2014a). More recently, there is an increasing awareness of the adverse effects of various mycotoxins on vulnerable structures in the intestines and the impairment of intestinal integrity (Bouhet and Oswald 2005; Grenier and Applegate 2013; Maresca et al. 2008; Pinton and Oswald 2014). A compromised barrier function is associated with an increased epithelial permeability and translocation of luminal allergens and pathogens, as well as a non-specific inflammatory response and an overstimulation of the gut-associated immune system (DeMeo et al. 2002; Groschwitz and Hogan 2009; Odenwald and Turner 2013; Pastorelli et al. 2013). The most prominent example of a mycotoxin primarily associated with an impairment of the intestinal integrity is deoxynivalenol, a trichothecene, which first had been recognized for its pro-inflammatory and immunomodulatory activities (Pestka et al. 1990; Pestka 2010a, b; Rotter et al. 1996). However, various other mycotoxins have been studied regarding their effects on the intestinal barrier, both in vitro as well as in vivo. The current review aims to provide a summary and discussion of the available evidence regarding direct effects of various mycotoxins on individual structures of the intestinal epithelial barrier. The mycotoxins addressed include the aflatoxins, zearalenone, ochratoxins, fumonisins and patulin, as well as T-2/HT-2 toxin, nivalenol and deoxynivalenol, as representatives of the class of the trichothecenes. The summary of the effects of individual mycotoxins on the intestinal barrier is preceded by a short introduction into common experimental models and test parameters to measure intestinal integrity.

## Experimental models used to assess intestinal permeability

### The Caco-2 cell model

During the last few decades, the use of different intestinal epithelial cell lines from various animal species as well as from human origin has been used to assess the effects of drugs and toxins on the permeability of the intestinal epithelium. Among them, the Caco-2 cell line (ATCC^®^ number: HTB-37), originally isolated from a human colon adenocarcinoma, is well accepted as a reference model to conduct transport studies as well as to investigate the effects on barrier function. Caco-2 cells are routinely cultivated as monolayers on permeable filters. During culturing, they undergo spontaneous differentiation resulting in polarization and formation of the tight junction (TJ) proteins between adjacent cells. Differentiated Caco-2 cells form polarized apical/mucosal and basolateral/serosal membranes that are impermeable and are structurally and functionally similar to epithelial cells of the small intestine (Artursson et al. 2012; Hidalgo et al. 1989; Sambuy et al. 2005; Sun and Pang 2007). A major advantage of the common technique to grow Caco-2 cells on transwell inserts is the fact that transport of drugs and toxins from the apical to the basolateral compartment can be measured. In turn, the established cell monolayer can be challenged from the apical (luminal) site as well as the basolateral site with toxins as well as other antigens and allow a wide range of functional parameters to be measured (Shimizu 2010; Sun et al. 2008; Sun and Pang 2007). In addition, this Caco-2 cell system is a commonly used model to study the rate of absorption and excretion of mycotoxins across the intestinal epithelium (Berger et al. 2003; Caloni et al. 2005, 2006; Pfeiffer et al. 2011; Schrickx et al. 2006; Tep et al. 2007; Videmann et al. 2007, 2009). Although the epithelial permeability for individual mycotoxins is beyond the scope of this review, the transepithelial transport of different mycotoxins has been briefly described and summarized in Electronic Supplementary Material, Table 1.

**Table 1:** Modulation of the intestinal barrier function by aflatoxins

Model	Concentration and exposure time	Effects on barrier function	References
*Aflatoxin*			
Caco-2 cells	150 µM 72 h	AFB_1_: decrease in TEER values	Gratz et al. (2007)
Caco-2 cells	1–100 µM 7 days	AFB_1_: decrease in TEER values Decrease in transcript level of CLDN3 and OCLN	Romero et al. (2016)
Broiler chicken	1.5 mg/kg bw 20 days	AFB_1_: increase in transcript level of CLDN1 and CLDN2 Increase in the plasma lactulose to rhamnose ratio	Chen et al. (2016)
Caco-2 cells	3.2–33 nM 24 h	AFM_1_: decrease in TEER values	Caloni et al. (2012)

### Measurement of transepithelial electrical resistance (TEER)

TEER is the first parameter measured to evaluate the integrity of the epithelial barrier in the Caco-2 cell model. A simple voltmeter device equipped with a pair of chopstick-like electrodes quantifies ion movement across a monolayer and is considered as an effective indicator for the developing barrier function. TEER is generally used to follow the cell differentiation process, and standard values for a completed non-permeable barrier are established based on individual devices and insert sizes. Even though TEER measurement is quick and easy and can be repeated as needed, it remains a non-specific endpoint. Routine TEER measurement is used to control the integrity of the epithelial layer in an experimental setting and as a first indicator of toxin-induced damages. However, no specific mechanisms and transport processes can be attributed to changes in TEER without further investigations. In comparison with the standard TEER assay, real-time cell electronic sensing was further developed. This technique is based on the continuous recording of cellular horizontal impedance, which enables a real-time monitoring of the integrity of the epithelial barrier and the potential effects of toxins and other agents that affect barrier integrity (Abassi et al. 2009; Akbari et al. 2014; Benson et al. 2013; Sun et al. 2012).

### Paracellular tracer flux assays

In addition to TEER measurement, determination of the paracellular flux of marker substances across the cell monolayer can be monitored (De Walle et al. 2010). These markers differ in size and need to be non-toxic, non-charged and water soluble, and they should neither be absorbed, nor metabolized by the cells (Arrieta et al. 2006; Bjarnason et al. 1995). The most common paracellular markers used in in vitro models are fluorescent compounds (such as lucifer yellow, LY) or fluorescently labeled compounds (such as fluorescein isothiocyanate (FITC)-dextran and FITC-inulin) (Jimison et al. 2012). In particular, apical-to-basolateral flux of paracellular markers is used to identify a compromised intestinal barrier function (Bischoff et al. 2014).

Paracellular tracer transport can also be measured in in vivo models by testing the presence of macromolecular tracers in the blood (such as FITC-dextran) after oral gavage. In addition, site-specific permeability alongside the gastrointestinal (GI) tract can be assessed by measuring the presence of a variety of small saccharide probes and/or chromium-labeled ethylenediaminetetraacetic acid (Cr-EDTA) in the urine of humans and experimental animals after oral administration. For example, sucrose and lactulose/mannitol are useful probes for determining permeability characteristics of the gastroduodenal region and the entire small intestine, while sucralose and Cr-EDTA are used to evaluate colonic permeability (Arrieta et al. 2006; Bjarnason et al. 1995; Meddings and Gibbons 1998).

### Assessment of the expression of TJ proteins

**Fig. 1:**
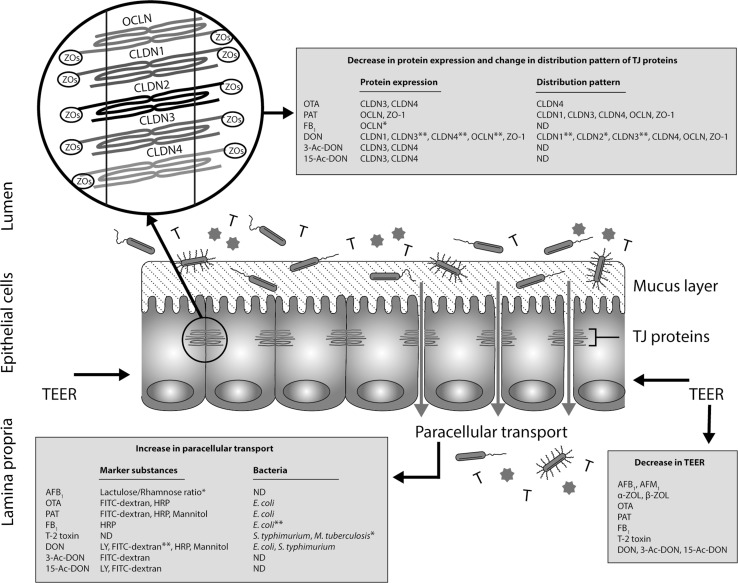
Schematic illustration of the mycotoxin-induced intestinal epithelial barrier breakdown. The gut mucosa is constantly challenged by a diverse microbial community (, ), food-borne toxins (T) and foreign antigens (). The most prominent examples of food-borne toxins primarily associated with an impairment of the intestinal barrier are mycotoxins. Various mycotoxins have been shown to induce intestinal barrier breakdown demonstrated by a decrease in TEER, an increase in paracellular transport and changes in the expression as well as distribution pattern of different TJ proteins. The data shown in the figure have been demonstrated by in vitro studies unless otherwise stated (*in vivo studies, **in vitro as well as in vivo studies). Abbreviations used: *3-Ac-DON* 3-acetyl deoxynivalenol, *15-Ac-DON* 15-acetyl deoxynivalenol, *AFB*_*1*_ aflatoxin B_1_, *AFM*_*1*_ aflatoxin M_1_, *α-ZOL* alpha-zearalenol, *β-ZOL* beta-zearalenol, *CLDNs* claudins, *DON* deoxynivalenol, *E. coli* *Escherichia coli*, *FB*_*1*_ fumonisin B_1_, *FITC-dextran* fluorescein isothiocyanate-dextran, *HRP* horseradish peroxidase, *LY* lucifer yellow, *M. tuberculosiss* *Mycobacterium tuberculosiss*, *ND* not determined, *OCLN* occludin, *OTA* ochratoxin A, *PAT* patulin, *S. typhimurium* *Salmonella typhimurium*, *TEER* transepithelial electrical resistance, *TJ* tight junction, *ZOs* zonula occludens

The major functional elements of the epithelial barrier are the TJ proteins, sealing the intercellular space between adherent epithelial cells (Groschwitz and Hogan 2009; Peterson and Artis 2014). TJs form an anastomosing network near the luminal surface, thus preventing a paracellular transport of luminal antigens (Fig. 1). TJs are composed of: I) transmembrane proteins whose extracellular domains cross the plasma membrane and interact with their partners on the adjacent cells and II) cytoplasmic scaffolding proteins, which are entirely located on the intracellular side of the plasma membrane. Transmembrane TJs form a horizontal barrier at the apical-lateral membrane of epithelial cells and consist of occludin (OCLN), claudins (CLDNs), junctional adhesion molecules (JAMs) and tricellulin. The cytoplasmic scaffolding proteins, like zonula occludens (ZOs) proteins, provide a direct link between transmembrane TJ proteins and the actin cytoskeleton (Chiba et al. 2008; Schneeberger and Lynch 2004; Tsukita et al. 2001). Increased TJ mRNA expression can indicate ongoing repair mechanisms in an established epithelial cell monolayer (Akbari et al. 2014). However, the assessment of TJs should not be limited to the gene level, since mRNA amount does not necessarily predict the protein level (Schwanhausser et al. 2011; Vogel et al. 2010). For example, our study showed that following deoxynivalenol exposure, a decrease in the protein level of CLDNs could be observed, associated with an up-regulation of the mRNA level of CLDNs (Akbari et al. 2014). Therefore, for the interpretation of barrier damage, qPCR and Western blot analysis are generally performed in parallel to measure mRNA and protein levels of TJs, respectively. In addition, the visualization of the subcellular localization of TJs by immunostaining is an additional tool to identify intestinal barrier dysfunction. All these measurements can be taken in different in vitro cell culture models as well as in intestinal explants and in vivo models.

### Intestinal explant model

Next to cell culture models, intestinal explants have been introduced as a model to test intestinal integrity. The model is based on the long-term experience with intestinal specimen mounted in the so-called Ussing chambers for the study of nutrient absorption. For these studies, sheets of intestinal segments are mounted in Ussing chambers and maintained in complete explant culture medium gassed with 95 % O_2_ and 5 % CO_2_ and kept at 37 °C with or without shaking for the entire culture time (Kolf-Clauw et al. 2009). The major advantage of this model is that explants maintain the complex cellular community and intestinal architecture, and therefore, cell–cell interactions can be studied. Moreover, segment-specific responses can be monitored alongside the GI tract. The main limitation of intestinal explant is that the period of culture during which the morphology and function of cells is preserved is very short, limiting the possibility to study delayed or long-term effects (Kolf-Clauw et al. 2009, 2013; Randall et al. 2011).

### In vivo models

In addition to the above-described in vitro (cell culture) or ex vivo (explant) assays, several markers of intestinal integrity can be directly measured in vivo in comparable models. This includes the paracellular flux assays and the assessment of the expression of TJ proteins together with histological approaches that provide insight into changes in the intestinal architecture, but also into epithelial cell damage (Bischoff et al. 2014). Zonulin, as an example, is a physiological modulator of intercellular TJs, and an increase in zonulin levels in serum is associated with an impaired intestinal permeability (Fasano 2011, 2012). Moreover, to identify the intestinal epithelial damage, serum concentrations of intestinal fatty acid-binding protein (IFABP) can be evaluated (Furuhashi and Hotamisligil 2008; Pelsers et al. 2003), but both parameters have not been widely applied in the assessment of mycotoxins. In contrast, histological investigations describing the effects of mycotoxins on villus architecture, goblet cells and mucus production (Paneth cells) are among routine approaches to detect the presence and the extent of epithelial cell damage and intestinal integrity following the exposure to mycotoxins and other toxic agents in various animal species (Bischoff et al. 2014; Blikslager et al. 2007; Cheat et al. 2015; Pinton et al. 2015).

## Effects of mycotoxins on intestinal permeability

Figure 1 provides a comprehensive overview of the available evidence regarding direct effects of various mycotoxins on the intestinal epithelial barrier. The direct effect of aflatoxins, zearalenone, ochratoxin A, patulin, fumonisin B_1_, T-2/HT-2 toxin, nivalenol and deoxynivalenol are extensively explained and discussed in the following section.

### Aflatoxins

Aflatoxins are naturally occurring mycotoxins that are produced by various species of *Aspergillus*. The major aflatoxins commonly isolated from foods and feeds are aflatoxins B_1_, B_2_, G_1_ and G_2_ (Leong et al. 2012). Aflatoxin B_1_ (AFB_1_), considered as the most toxic form, is metabolized by liver cytochrome P450 (CYP) enzymes (mainly by CYP3A4 and CYP1A2) to an AFB_1_-8,9-exo-epoxide and AFB_1_-8,9-endo-epoxide. The exo-epoxide rapidly binds to DNA and forms the 8,9-dihydro-8-(N7-guanyl)-9-hydroxy AFB_1_ (AFB_1_-N7-Gua) adduct. If this DNA damage is not repaired before DNA replication, it causes mutational effects in the third base of codon 249 in the p53 tumor suppressor gene. P53 is the most frequently targeted gene in human carcinogenesis, with a mutation frequency of 50 % in most major cancers (Bedard and Massey 2006; Hamid et al. 2013; Wild and Turner 2002); hence, this mutation is considered as a key event in aflatoxin-induced carcinogenesis. The endo-epoxide primarily binds to cellular proteins and is associated with direct cytotoxicity and the impairment of liver function. AFB_1_ is classified as a group 1 carcinogen (carcinogenic to humans) by the International Agency for Research on Cancer (IARC) (IARC 2002). Epidemiological evidence suggest a synergistic effect of aflatoxin B_1_ and chronic hepatitis B virus infections in the prevalence of liver cancer in humans (Liu et al. 2012; Nordenstedt et al. 2010; Wild and Turner 2002; Wu and Santella 2012).

Another important hepatic metabolite of AFB_1_ is aflatoxin M_1_ (AFM_1_), which is excreted into milk both in animals and in humans. This results in an undesirable exposure of infants. AFM_1_ is less biologically active than AFB_1_, but can also be converted into an AFM_1_-epoxide that can bind to DNA and form a AFM_1_-N7-Gua which leads to hepatotoxicity and hepato-carcinogenicity (Egner et al. 2003; Leong et al. 2012; Marin et al. 2013). IARC has classified AFM_1_ as a group 2B carcinogen (possibly carcinogenic to humans) (IARC 2002).

### Effects of aflatoxins on intestinal barrier function

In consideration of the primary hepatotoxicity and hepato-carcinogenicity, only very few studies have been conducted showing that aflatoxins exposure might compromise also intestinal permeability (Table 1). Gratz et al. (Gratz et al. 2007) showed that AFB_1_ induces a time-dependent decrease in TEER values of Caco-2 cells. This effect was only observed at high concentrations and in the presence of activated CYP3A4, confirming the biotransformation-dependent toxicity of AFB_1_. Recently, it has been reported that the TEER decrease in AFB_1_-exposed Caco-2 cells at concentrations up to 100 µM for 7 days is accompanied with a decrease in transcript level of CLDN3 and OCLN, while the level of CLDN4 remained unaffected (Romero et al. 2016). Contradictory results have been observed in in vivo models, since Galarza-Seeber et al. observed that AFB_1_ does not increase gut permeability in broiler chickens, whereas a study conducted by Chen et al. clearly showed that AFB_1_ affects intestinal barrier function in broiler chickens as indicated by an increase in the plasma lactulose to rhamnose ratio (L/R ratio) as well as an increase in transcript level of CLDN1 and CLDN2 in the jejunum (Chen et al. 2016; Galarza-Seeber et al. 2016). It has been demonstrated that exposure to much lower AFM_1_ concentrations either to the apical or basolateral surface of the Caco-2 cell monolayer results in a slight, but significant TEER decrease (Caloni et al. 2012). The subcellular localization of OCLN and ZO-1 remained unaffected as observed by immunostaining. Further studies would be necessary to unravel the potential clinical impact of aflatoxins, in particular AFM_1_, on epithelial barrier integrity in infants.

### Zearalenone

Zearalenone (ZEA) is a non-steroidal estrogenic mycotoxin produced by various *Fusarium* species (Marin et al. 2013). Following oral exposure, absorbed ZEA is predominantly metabolized into alpha-zearalenol (α-ZOL) and beta-zearalenol (β-ZOL) by hepatic hydroxysteroid dehydrogenases (Kleinova et al. 2002; Malekinejad et al. 2005, 2006a, b; Warth et al. 2013). The reproductive system is the major target organ for ZEA and its metabolites, which are implicated in reproductive disorders and hyperestrogenic syndromes in animals and humans (Malekinejad et al. 2006b, 2007; Minervini and Dell’Aquila 2008; Schoevers et al. 2012; Zinedine et al. 2007). The main mechanism of action for the estrogenic effects of ZEA is the ability of this mycotoxin to bind and activate estrogenic receptors (ERs), in particular ERα and ERβ (Minervini and Dell’Aquila 2008; Takemura et al. 2007). In addition, ZEA is believed to induce cytotoxic, hepatotoxic, hematotoxic, immunotoxic and genotoxic effects, which are probably related to intracellular oxidative stress generated by ZEA leading to oxidative DNA damage and cellular apoptosis (Abid-Essefi et al. 2004; Hassen et al. 2007; Liu et al. 2014; Marin et al. 2011; Pfohl-Leszkowicz et al. 1995; Zinedine et al. 2007). DNA adduct formation induced by ZEA has been occasionally reported in in vitro as well as in vivo models (Abid-Essefi et al. 2003; Pfohl-Leszkowicz et al. 1995; Zinedine et al. 2007). However, IARC has classified ZEA as a group 3 carcinogen (not carcinogenic to humans) due to inadequate evidence for the carcinogenicity of ZEA in humans (IARC 1987).

### Effects of zearalenone on intestinal barrier function

It is known that ZEA can induce cytotoxic and apoptotic effects on human enterocytes (Abid-Essefi et al. 2003, 2004; Calvert et al. 2005; Kouadio et al. 2005); however, the effect of ZEA on intestinal barrier has not been extensively studied (Table 2). It has recently been reported that ZEA has no effect on TEER values of IPEC-1 cells, whereas exposure to α-ZOL and β-ZOL causes a dramatic decrease in TEER levels in a time-dependent manner (Marin et al. 2015). Another study has shown that the exposure of rats to ZEA (0.3–146 mg/kg bw) for 7 days results in a down-regulation of CLDN4 and OCLN mRNA expression in the jejunum (Liu et al. 2014). Further research is needed to extend the current knowledge of impairment of intestinal barrier function induced by ZAE as well as its metabolites.

**Table 2:** Modulation of the intestinal barrier function by zearalenone

Model	Concentration and exposure time	Effects on barrier function	References
*Zearalenone*			
IPEC-1 cells	25–50 µM 10 days	ZEA: no effect on TEER values α-ZOL and β-ZOL: decrease in TEER values	Marin et al. (2015)
Rat	0.3–146 mg/kg bw 7 days	ZEA: decrease in transcript level of CLDN4 and OCLN in jejunum	Liu et al. (2014)

### Ochratoxin A

Ochratoxin A (OTA) is a mycotoxin produced by various species of *Aspergillus* and *Penicillium* (Marin et al. 2013). Exposure to OTA is a worldwide phenomenon, as evidenced by the presence of OTA in the majority of the tested human blood samples in many countries (Peraica et al. 2001; Pfohl-Leszkowicz and Manderville 2007; Studer-Rohr et al. 2000). The kidney is the major target organ for OTA and its derivatives, and some epidemiological studies in humans have associated the exposure to OTA with a chronic tubulo-interstitial nephritis (also denoted Balkan endemic nephropathy (BEN)) and urothelial tract tumors (Fink-Gremmels 2005; Grollman and Jelakovic 2007; Marin et al. 2013). At higher concentrations, OTA has been shown to be nephrotoxic, teratogenic and immunotoxic. IARC has classified OTA as a group 2B carcinogen (possibly carcinogenic to humans) on the basis of sufficient evidence for carcinogenicity in animal studies (IARC 2002). A number of mechanisms are described to be involved in OTA toxicity, including (1) inhibition of protein synthesis through inhibition of phenylalanyl-tRNA synthetase, (2) mitochondrial dysfunctions and the production of reactive oxygen and nitrogen species (ROS and RNS) and lipid peroxidation, (3) inhibition of histone acetyltransferase, which leads to disruption of mitosis and chromosomal instability as well as, (4) DNA adducts, particularly deoxyguanosine (dG) adducts (Fink-Gremmels et al. 1995; Mally 2012; Omar et al. 1990; Pfohl-Leszkowicz and Manderville 2012; Sorrenti et al. 2013). Although the kidney is generally believed to be the main target organ for OTA toxicity, its well-known inhibition of cellular protein synthesis and the generation of reactive oxygen as well as nitrogen species suggest that the liver and the GI tract may be a possible target organ for OTA as well (Bouhet and Oswald 2005; Grenier and Applegate 2013).

### Effects of ochratoxin A on intestinal barrier function

Modulation of the intestinal barrier by OTA has mainly been studied using the in vitro Caco-2 cell model (Table 3). For the first time, Maresca et al. (2001) showed that the OTA exposure results in a concentration- and time-dependent decrease in TEER values of both Caco-2 and HT-29-D4 cells. They showed that the apical surface is more susceptible to OTA in comparison with exposure via the basolateral surface; in contrast, other studies reported that both apical and basolateral surfaces are equally affected by OTA (Maresca et al. 2001; McLaughlin et al. 2004). Ranaldi et al. (Ranaldi et al. 2007) found that the TEER decrease in OTA-exposed Caco-2 cells at concentrations up to 200 µM for 48 h is reversible and a full recovery of TEER value is achieved within 24 h after cessation of mycotoxin exposure. It has been reported that the OTA-induced TEER decrease is accompanied with an increase in the translocation of paracellular markers, such as 4, 10 kDa FITC-dextran and horseradish peroxidase (HRP, ~44 kDa) (Table 3). The OTA-induced permeability is shown to be size selective, since translocation of 20 and 40 kDa FITC-dextran remains unchanged after exposure of Caco-2 cells to OTA up to 100 µM for 24 h (Maresca et al. 2008; McLaughlin et al. 2004). OTA-induced intestinal permeability is associated with specific alterations in the expression (at transcriptional and protein levels) as well as distribution of different TJs. A down-regulation in mRNA expression levels of CLDN3, CLDN4 and OCLN was observed in OTA-exposed Caco-2 cells (Romero et al. 2016). CLDN3 and CLDN4 have been reported to be the most susceptible TJ proteins regarding OTA exposure to human intestinal epithelial cells (Lambert et al. 2007; McLaughlin et al. 2004; Ranaldi et al. 2007; Romero et al. 2016). Of clinical relevance is the finding that the OTA-induced intestinal barrier impairment in concentrations equal or higher than 1 µM OTA triggers a concentration- and time-dependent increase in the translocation of *Escherichia coli* across Caco-2 cell monolayers (Maresca et al. 2008).

**Table 3:** Modulation of the intestinal barrier function by ochratoxin A

Model	Concentration and exposure time	Effects on barrier function	References
*Ochratoxin A*			
Caco-2 cells	100 µM 24 h	Decrease in TEER values Increase in permeability of 4 and 10 kDa FITC-dextran Decrease in protein expression of CLDN3 and CLDN4	McLaughlin et al. (2004)
Caco-2 cells	1–100 µM 12 h	Decrease in TEER values Increase in permeability of HRP and 4 kDa FITC-dextran Increase in translocation of commensal *Escherichia coli* (strain k12)	Maresca et al. (2008)
Caco-2 cells	100 µM 24 h	Decrease in TEER values Decrease in protein expression of CLDN3 and CLDN4	Lambert et al. (2007)
Caco-2 cells	40–1000 µM 48 h	Decrease in TEER values Affect the distribution pattern of CLDN4	Ranaldi et al. (2007)
Caco-2 cells	10 µM 3 h	Neither a significant decrease in TEER values nor an increase in permeability of [^14^C]-mannitol	Sergent et al. (2005)
Caco-2 cells	1–100 µM 7 days	Decrease in TEER values Decrease in transcript level of CLDN3, CLDN4 and OCLN	Romero et al. (2016)
Caco-2 cells HT-29-D4 cells	00.1–100 µM 48 h	Decrease in TEER values	Maresca et al. (2001)

### Patulin

Patulin (PAT) is a mycotoxin produced by various species of *Penicillium*, *Aspergillus* and *Byssochylamys*, known as fruit-spoiling fungi (Marin et al. 2013; Moake et al. 2005). Based on experimental models in mice, PAT was initially suspected to increase the prevalence of gastric cancers, but the IARC has classified PAT as a group 3 carcinogen (not carcinogenic to humans) due to inadequate evidence for the carcinogenicity of PAT in both experimental animals and humans (IARC 1987). Currently, the GI tract and the immune system are thought to be the most affected tissues following PAT exposure (Maresca and Fantini 2010; Moake et al. 2005). PAT is believed to induce cytotoxicity by forming covalent adducts with essential cellular thiols (organic compounds that contain a sulfhydryl group), by which it inhibits the activity of many enzymes. One of the most likely cellular targets of PAT is the sulfhydryl group of cysteine (Cys) and glutathione (GSH) leading to depletion of glutathione and subsequent increased generation of ROS (Fliege and Metzler 2000; Marin et al. 2013; Pfenning et al. 2014; Puel et al. 2010; Schebb et al. 2009). A recent study conducted by Boussabbeh et al. (Boussabbeh et al. 2015) revealed that PAT induces apoptosis through the ROS-mediated endoplasmic reticulum stress pathway.

### Effects of patulin on intestinal barrier function

Impairment of intestinal barrier integrity induced by PAT has been clearly shown in different studies (Table 4). PAT is found to induce a rapid and dramatic decrease in TEER values of Caco-2 and HT-29-D4 monolayers (Assuncao et al. 2014, 2016; Mahfoud et al. 2002; McLaughlin et al. 2009). PAT exposure to either apical or basolateral surface resulted in a concentration- and time-dependent decrease in TEER levels. The apical surface seems to be slightly more sensitive than the basolateral surface (Mahfoud et al. 2002; McLaughlin et al. 2009). Mohan et al. (2012) showed that PAT at concentrations equal or higher than 1.6 µM causes an increase in plasma membrane permeability, observed by increased TOTO-3 fluorescence intensity. In addition, it has been reported that PAT induces the permeability of different paracellular markers such as HRP and FITC-dextrans of 4–40 kDa across the intestinal epithelium (Table 4) (Katsuyama et al. 2014; Maresca et al. 2008; McLaughlin et al. 2009). Maresca et al. (2008) showed that the impairment of intestinal integrity by PAT results in an increased translocation of *E. coli* across Caco-2 cell monolayers. There are different studies demonstrating specific effects of PAT on TJs. For example, it is shown that 5-h exposure of Caco-2 cell monolayers to 100 µM PAT leads to proteolytic cleavage of OCLN and a significant reduction in ZO-1 protein levels. However, the expression levels of CLDN1, CLDN3 and CLDN4 were not changed (McLaughlin et al. 2009). Kawauchiya et al. (2011) demonstrated that the exposure of Caco-2 cells to 50 µM PAT resulted in a gradual decrease in protein expression of ZO-1, while the expression levels of CLDN4 and OCLN remained unaffected up to 72 h. Interestingly, the decreased ZO-1 expression observed in latter study was correlated with an increased phosphorylation of this protein, while phosphorylation of CLDN4 and OCLN was not detected. This is in contrast to the finding of Katsuyama et al. (2014) who reported an increase in phosphorylation of CLDN4 following a 24-h exposure of Caco-2 cells to PAT at a concentration of 50 µM. PAT also affects the distribution pattern of different TJs including CLDN1, CLDN3, CLDN4, OCLN and ZO-1 (Katsuyama et al. 2014; Kawauchiya et al. 2011; McLaughlin et al. 2009). Moreover, PAT exposed to isolated rat colonic mucosa at a concentration of 500 µM for 2 h has been shown to induce intestinal barrier breakdown demonstrated by a decrease in TEER values and an increase in [^14^C]-mannitol (182 Da) permeability (Mohan et al. 2012). There are a few possible mechanisms underlying the PAT-induced impairment of TJs and intestinal barrier function. For example, Mahfoud et al. (2002) showed that the PAT-induced TEER decrease involves inhibition of protein tyrosine phosphatase (PTP) through inactivation of cysteine residues in the catalytic domains of PTP. The high affinity of PAT for sulfhydryl groups of Cys and GSH (explained above) may account for the barrier impairment, since addition of 1.6 mM GSH was found to completely prevent PAT-induced TEER drop. Therefore, higher amount of GSH in Caco-2 cells compared to HT-29-D4 cells (8.0 vs. 4.5 nmol/mg of protein) may explain the different sensitivity of these cells to PAT (Mahfoud et al. 2002). It has been recently suggested that PAT decreases the expression of density-enhanced phosphatase-1 (DEP-1) through down-regulation of proliferator-activated receptor gamma (PPARγ) (Katsuyama et al. 2014). DEP-1 is a class III transmembrane phophatidyl-inositol-phosphate, which has been proposed to regulate different signal transduction pathways, such as cell migration, proliferation, differentiation and adhesion (Balavenkatraman et al. 2006; Petermann et al. 2011). Furthermore, it has been observed that a PAT-mediated decrease in DEP-1 results in hyperphosphorylation of CLDN4 and subsequently hinders the interaction between ZO-1 and CLDN4, which leads to release of CLDN4 from the TJ network (Katsuyama et al. 2014). In addition, McLaughlin et al. (McLaughlin et al. 2009) speculated that matrix metalloproteinases (MMPs) may play a role, at least partly, in the observed intestinal barrier impairment induced by PAT, since inhibition of MMP partially protected OCLN from PAT-mediated cleavage. However, according to their findings, the reduction in ZO-1 levels is not prevented by MMP inhibitors.

**Table 4:** Modulation of the intestinal barrier function by patulin

Model	Concentration and exposure time	Effects on barrier function	References
*Patulin*			
Caco-2 cells	100 µM 5 h	Decrease in TEER values Increase in permeability of 4, 10, 20 and 40 kDa FITC-dextran Proteolysis of OCLN Decrease in protein expression of ZO-1 Affect the distribution pattern of CLDN1, CLDN3, CLDN4, OCLN and ZO-1	McLaughlin et al. (2009)
Caco-2 cells	50 µM 72 h	Decrease in TEER values Decrease in protein expression of ZO-1 Increase in phosphorylation of ZO-1 Affect the distribution pattern of CLDN4, OCLN and ZO-1	Kawauchiya et al. (2011)
Caco-2 cells	50 µM 36 h	Decrease in TEER values Increase in permeability of 4 kDa FITC-dextran Increase in phosphorylation of CLDN4 Affect the distribution pattern of ZO-1	Katsuyama et al. (2014)
Caco-2 cells	1–100 µM 12 h	Decrease in TEER values Increase in permeability of HRP and 4 kDa FITC-dextran Increase in translocation of commensal *Escherichia coli* (strain k12)	Maresca et al. (2008)
Caco-2 cells	0.2–100 µM 72 h	Increase in plasma membrane permeability	Mohan et al. (2012)
Caco-2 cells	25 nM–95 µM 24 h	Decrease in TEER values	Assuncao et al. (2014)
Caco-2 cells	50 µM 24 h	Decrease in TEER values Decrease in protein expression of ZO-1	Assuncao et al. (2016)
Caco-2 cells HT-29-D4 cells	1–100 µM 24 h	Decrease in TEER values	Mahfoud et al. (2002)
Rat colonic explants	100–500 µM 2 h	Decrease in TEER values Increase in permeability of [^14^C]-mannitol	Mohan et al. (2012)

### Fumonisin B_1_

Fumonisin B_1_ (FB_1_) is the major representative of structurally related fumonisins produced by various specious of *Fusarium,* predominantly by *Fusarium verticillioidis.* Initially, FB_1_ has been associated with an increased prevalence of esophageal cancers in humans in the Transkei region of South Africa (Chu and Li 1994; Rheder et al. 1992; Sydenham et al. 1990). IARC has classified FB_1_ as a group 2B carcinogen (possibly carcinogenic to humans) (IARC 2002). Maternal exposure to fumonisins increases the risk of neural tube defects (such as spina bifida and anencephaly) in offspring, mainly through interference with the function of folate-binding protein and the utilization of folic acid (Gelineau-van Waes et al. 2005; Marasas et al. 2004; Missmer et al. 2006; Sadler et al. 2002). FB_1_ has been shown to be hepatotoxic, nephrotoxic, carcinogenic and immunotoxic in various animal species (Voss et al. 2007). The main mechanism of action is inhibition of the enzyme ceramide synthase (CerS) (Enongene et al. 2000; Loiseau et al. 2007, 2015; Voss et al. 2007). CerS is a key enzyme that catalyzes the formation of complex sphingolipids from the sphingoid bases (Mullen et al. 2012; Voss et al. 2007). FB_1_ is observed to inhibit mainly CerS4 isomers (CerS1 and CerS2 isomers are also inhibited to a lesser extent) and leads to the accumulation of sphingoid bases (including sphinganine and sphingosine) and in turn to a depletion of ceramide and complex sphingolipids (Enongene et al. 2000; Loiseau et al. 2007, 2015; Marin et al. 2013; Voss et al. 2007). It is well known that sphingolipids participate in a variety of cellular signaling pathways, such as regulation of cell proliferation, differentiation and apoptosis (Mullen et al. 2012; Ribeiro et al. 2010). Although liver and kidney are thought to be the most affected tissues by FB_1_ in animal species, the GI tract has also been reported as a possible target organ for FB_1_ (Bouhet and Oswald 2007; Voss et al. 2007). It has been shown that a single subcutaneous injection of FB_1_ (25 mg/kg body weight (bw)) causes a transient increase in sphinganine and sphingosine in the mouse small intestine over 24 h (Enongene et al. 2000). Exposure of pigs to FB_1_ (1.5 mg/kg bw) for 7 days results in a significant increase in the concentration of sphinganine and sphingosine and a decrease in the total glycolipid content as well as alteration in the jejunal glycolipid composition, whereas no changes are observed in the duodenum and ileum (Loiseau et al. 2007).

### Effects of fumonisin B_1_ on intestinal barrier function

Impairment of the intestinal barrier integrity induced by FB_1_ has been shown in different in vitro, ex vivo and in vivo studies (Table 5). A concentration- and time-dependent decrease in TEER values of Caco-2 and IPEC-1 cells (intestinal porcine epithelial cells) have been observed after FB_1_ exposure; however, this process in IPEC-1 cells was time dependent as significant effects occurred only after long-term exposure (Bouhet et al. 2004; Loiseau et al. 2007; Romero et al. 2016). Bouhet et al. (2004) demonstrated that FB_1_-induced decrease in TEER values is independent from the differentiation stage of IPEC-1 cells and this TEER drop is partially reversible. Another study has shown that the impaired intestinal barrier results in an increase in permeability for FB_1_ across the IPEC-1 cells, suggesting that after long-term exposure, the very low absorption rate (normally ~3 %) may increase over time (Loiseau et al. 2007). Surprisingly, an increase in TEER values of porcine jejunal explants is reported after 2-h exposure to FB_1_ at concentration of 10 µM. In contrast, a significant increase in HRP permeability is reported following treatment with 10 µM FB_1_ over the same time period (Lalles et al. 2009). In vitro and in vivo studies have showed that FB_1_-induced barrier function impairment causes an increase in the translocation of pathogenic *E. coli* across intestinal epithelial cells (Bouhet and Oswald 2007; Oswald et al. 2003). Furthermore, *E. coli* could be recovered from lung and mesenteric lymph nodes 7 days after oral exposure of pigs to FB_1_ at a dose of 0.5 mg/kg bw (Oswald et al. 2003). The FB_1_-induced barrier impairment in Caco-2 cells has been shown to be accompanied with a significant decrease in the transcript level of CLDN3, CLDN4 and OCLN (Romero et al. 2016). Bracarense et al. (Bracarense et al. 2012) observed that the exposure of piglets to a FB_1_-contaminated diet (3 mg/kg) for 5 weeks significantly decreases the protein expression of OCLN in ileum. Further studies would be needed to clarify the involvement of TJ impairment in FB_1_-induced impairment of the intestinal integrity.

**Table 5:** Modulation of the intestinal barrier function by fumonisin B_1_

Model	Concentration and exposure time	Effects on barrier function	References
*Fumonisin B*_*1*_			
Caco-2 cells	1–100 µM 7 days	Decrease in TEER values Decrease in transcript level of CLDN3, CLDN4 and OCLN	Romero et al. (2016)
IPEC-1 cells	50–200 µM 16 days	Decrease in TEER values Increase in permeability of FB_1_	Loiseau et al. (2007)
IPEC-1 cells	20–200 µM 4 h	Increase in translocation of pathogenic *Escherichia coli* (strain 28C)	Bouhet and Oswald (2007)
IPEC-1 cells	50–500 µM 28 days	Decrease in TEER values	Bouhet et al. (2004)
Porcine jejunal explants	10 µM 2 h	Increase in TEER values Increase in permeability of HRP	Lalles et al. (2009)
Piglet	3 mg/kg feed 5 weeks	Decrease in protein expression of OCLN in ileum	Bracarense et al. (2012)
Piglet	0.5 mg/kg bw 7 days	Increase in translocation of pathogenic *Escherichia coli* (strain 28CNal^r^)	Oswald et al. (2003)

### Trichothecenes

The class of trichothecenes comprises a unique family of over 200 tetracyclic sesquiterpenoid fungal metabolites produced by various species of the genera *Fusarium*, *Stachybotrys, Cephalosporium,* *Myrothecium, Spicellum*, *Verticimonosporium, Trichoderma* and *Trichothecium* (McCormick et al. 2011; Rocha et al. 2005; Wu et al. 2013). Common structure elements of trichothecenes are a C-9, C-10 double bond and C-12, C-13 epoxide moiety contributing to the toxicity of trichothecenes (Escriva et al. 2015; McCormick et al. 2011; Sudakin 2003). Trichothecenes are classified into four different types (type A-D) according to the characteristic functional group. Type A trichothecenes are characterized by a hydroxyl motif at C-8 (e.g., T-2/HT-2 toxins), whereas type B trichothecenes carry a keto (carbonyl) motif at this position (e.g., nivalenol, deoxynivalenol). Type C trichothecenes have an additional epoxide group at the C-7, C-8 or C-9, C-10 position (e.g., crotocin), while type D trichothecenes possess a macrocyclic ring between the C-4, C-15 positions (e.g., roridin) (McCormick et al. 2011; Rocha et al. 2005; Shank et al. 2011; Sudakin 2003; Wu et al. 2013). Among them, type A and type B are known to be the most prevalent trichothecenes (Nathanail et al. 2015; Wu et al. 2013). At the cellular level, type A and type B trichothecenes not only interact with the peptidyl transferase at the 60S ribosomal subunit to cause a translational arrest and protein synthesis inhibition, but also activate intracellular protein kinases, particularly mitogen-activated protein kinases (MAPKs) and their downstream effectors resulting in a process, known as ribotoxic stress response (Rocha et al. 2005; Shank et al. 2011; Wu et al. 2013, 2014b). Rapidly dividing cells, particularly intestinal epithelial cells and immune cells, are generally believed to be the major target organs for type A and type B trichothecenes (Li et al. 2011; Pinton et al. 2012). In consideration of the complex group of trichothecenes and the availability of detailed investigations, only major representatives of this class of mycotoxins such as T-2/HT-2 toxin, nivalenol (NIV) and deoxynivalenol (DON) will be discussed in more detail below. Their effects on intestinal barrier integrity are summarized in Table 6 at the end of this chapter.

**Table 6:** Modulation of the intestinal barrier function by trichothecenes

Model	Concentration and exposure time	Effects on barrier function	References
*T-2 toxin*			
Caco-2 cells	1–100 µM 7 days	Decrease in TEER values Decrease in transcript level of CLDN3, CLDN4 and OCLN	Romero et al. (2016)
IPEC-J2 cells	0.21–210 nM 72 h	Decrease in TEER values Increase in permeability of doxycycline and paromomycin	Goossens et al. (2012)
IPEC-J2 cells	1.6–10.7 nM 1 h	Increase in translocation of *Salmonella typhimurium* (strain 112910a)	Verbrugghe et al. (2012)
Mouse	3.3 mg/kg bw 20 days	Increase in translocation of *Mycobacterium tuberculosis* (strain H37RvR-KM)	Kanai and Kondo (1984)
*Deoxynivalenol*			
Caco-2 cells	1.39–12.5 µM 24 h	Decrease in TEER values Decrease in horizontal impedance value Increase in permeability of LY and 4 kDa FITC-dextran Increase in transcript level of CLDN3, CLDN4, OCLN and ZO-1 Decrease in protein expression of CLDN1, CLDN3 and CLDN4 Affect the distribution pattern of CLDN1, CLDN3, CLDN4, OCLN and ZO-1	Akbari et al. (2014)
Caco-2 cells	0.16–16 µM 24 h	Decrease in TEER values Increase in permeability of mannitol Increase in transcript level of CLDN4 and OCLN Decrease in protein expression of CLDN4	De Walle et al. (2010)
Caco-2 cells	5–100 µM 48 h	Decrease in TEER values Increase in permeability of 4 kDa FITC-dextran Increase in translocation of pathogenic *Escherichia coli* (strain 28C) Decrease in protein expression of CLDN4	Pinton et al. (2009)
Caco-2 cells	1–100 µM 12 h	Decrease in TEER values Increase in permeability of HRP and 4 kDa FITC-dextran Increase in translocation of commensal *Escherichia coli* (strain k12)	Maresca et al. (2008)
Caco-2 cells	0.37–1.5 µM 6–120 h	Decrease in horizontal impedance value of undifferentiated cells	Manda et al. (2015)
Caco-2 cells T84 cells	0.16–0.67 µM 14 days	Decrease in TEER values Increase in permeability of LY	Kasuga et al. (1998)
HT-29-D4 cells	0.001–100 µM 48 h	Decrease in TEER values	Maresca et al. (2002)
IPEC-1 cells	30 µM 48 h	Decrease in TEER values Increase in permeability of 4 kDa FITC-dextran Decrease in protein expression of CLDN4 Affect the distribution pattern of CLDN4	Pinton et al. (2010)
IPEC-1 cells	5–50 µM 48 h	Decrease in TEER values Increase in permeability of 4 kDa FITC-dextran Decrease in protein expression of CLDN3 and CLDN4	Pinton et al. (2009)
IPEC-1 cells IPEC-J2 cells	0.67–6.7 µM 48 h	Decrease in protein expression of ZO-1 Affect the distribution pattern of ZO-1	Diesing et al. (2011b)
IPEC-J2 cells	6.74 µM 48 h	Decrease in TEER values Decrease in protein expression of CLDN3, OCLN and ZO-1 Affect the distribution pattern of ZO-1	Gu et al. (2014)
IPEC-J2 cells	0.67–13.4 µM 24–72 h	Decrease in TEER values Decrease in protein expression of CLDN3 and ZO-1 Affect the distribution pattern of CLDN3	Diesing et al. (2011a)
IPEC-J2 cells	1.68–33.7 µM 72 h	Decrease in TEER values Increase in permeability of doxycycline and paromomycin	Goossens et al. (2012)
IPEC-J2 cells	0.33–3.3 µM 24 h	Increase in translocation of pathogenic *Salmonella typhimurium* (strain 112910a)	Vandenbroucke et al. (2011)
IPEC-J2 cells	4 µM 12 h	Decrease in TEER values Increase in permeability of 4 kDa FITC-dextran Increase in translocation of commensal *Escherichia coli* (strain ATCC^®^ 25922™) Increase in transcript level of CLDN1, CLDN4, OCLN and ZO-1 Decrease in protein expression of CLDN3 and CLDN4	Ling et al. (2016)
Porcine jejunal explants	5–50 µM 2 h	Increase in permeability of 4 kDa FITC-dextran	Pinton et al. (2009)
Piglet	3 mg/kg feed 5 weeks	Decrease in protein expression of OCLN in ileum	Bracarense et al. (2012)
Pig	2.85 mg/kg feed 5 weeks	Decrease in protein expression of CLDN4 in jejunum	Pinton et al. (2009)
Pig	3.5 mg/kg feed 6 weeks	Decrease in transcript level of CLDN3, CLDN4 and OCLN in ileum	Lessard et al. (2015)
Pig	0.9 mg/kg feed 10 days	Increase in transcript level of CLDNs (cecum), OCLD (duodenum, ileum, cecum and colon) and ZOs (duodenum and colon) Decrease in transcript level of CLDN4, OCLN, ZO-1 and ZO-2 in jejunum Increase in protein expression of OCLN in duodenum, jejunum and colon	Alizadeh et al. (2015)
Mouse	25 mg/kg bw 6 h	Increase in permeability of 4 kDa FITC-dextran Increase in transcript level of CLDN2, CLDN3 and CLDN4 in distal small intestine Affect the distribution pattern of CLDN1-3 in distal small intestine	Akbari et al. (2014)
Mouse	5 mg/kg bw 24 h	Increase in transcript level of CLDN2 and CLDN3 in duodenum Decrease in protein expression of CLDN3 in duodenum	Bol-Schoenmakers et al. (2016)
Broiler chicken	7.5 mg/kg feed 3 weeks	Increase in transcript level of CLDN5 in jejunum and CLDN1, CLDN5, ZO-1 and ZO-2 in ileum	Osselaere et al. (2013)
*3- and 15-acetyl deoxynivalenol*			
Caco-2 cells	3.37 µM 6 h	Decrease in TEER values 15-Ac-DON > DON > 3-Ac-DON Increase in permeability of LY 15-Ac-DON	Kadota et al. (2013)
IPEC-1 cells	10–30 µM 24–48 h	Decrease in TEER values Increase in permeability of 4 kDa FITC-dextran 15-Ac-DON > DON > 3-Ac-DON Decrease in protein expression of CLDN3 and CLDN4 15-Ac-DON > DON = 3-Ac-DON	Pinton et al. (2012)

### T-2/HT-2 toxin

Historically, prolonged exposure of humans to T-2 toxin has been associated with a disease known as alimentary toxic aleukia (ATA); characterized by nausea, vomiting, diarrhea, gastroenteritis, leukopenia (aleukia), hemorrhages, skin inflammation and in severe cases a death due to asphyxia (Joffe 1971). Genotoxicity and mutagenicity of T-2 are still a matter of controversial debate, and IARC has classified T-2 toxin as a group 3 carcinogen (not carcinogenic to humans) due to inadequate evidence for the carcinogenicity in both experimental animals and humans (IARC 1993). The major mechanisms of toxicity of T-2 toxin are described as I) inhibition of protein synthesis (at the initiation step of protein translation) through interaction with the peptidyl transferase at the 60S ribosomal subunit and II) generation of ROS and oxidative stress leading to caspase-mediated cellular apoptosis (Chaudhari et al. 2009; Rocha et al. 2005; Wu et al. 2013, 2014b). Rapidly after ingestion, T-2 toxin is mainly metabolized into HT-2 toxin, through a deacetylation reaction by intestinal microflora, and various hydroxylated metabolites in the liver. The toxicity of HT-2 is quite similar to that of the T-2 toxin, and their effects cannot be differentiated. However, it has been speculated that HT-2 toxin is responsible for the observed in vivo toxicity following T-2 toxin ingestion (Escriva et al. 2015; Li et al. 2011; Wu et al. 2013).

### Effects of T-2/HT-2 toxin on intestinal barrier function

Despite the well-documented clinical and pathological intestinal lesions induced by T-2 toxin (Pinton et al. 2012), the effects of T-2 toxin on intestinal integrity have hardly been studied (Table 6). However, a study conducted by Goossens et al. (2012) clearly showed that T-2 toxin causes an impairment of the barrier function at a concentration of 21 nM as observed by a decrease in TEER values and an increase in the passage of the antibiotics doxycycline and paromomycin across IPEC-J2 cells (intestinal porcine epithelial cells). Another study reported that the exposure of mice to T-2 toxin (3.3 mg/kg bw) for 20 days results in an increased translocation of *Mycobacterium tuberculosis* (Kanai and Kondo 1984). In addition, a significant increase in the translocation of *Salmonella typhimurium* across IPEC-J2 cell monolayer occurs already 30 min after T-2 toxin exposure with concentrations as low as 2.1 nM (Verbrugghe et al. 2012). Surprisingly in the same study, TEER values remained unaffected up to 24 h after exposure to concentrations of T-2 toxin ranging from 1.6 to 10.7 nM (Verbrugghe et al. 2012). It has recently been reported that the TEER decrease in T-2 toxin-exposed Caco-2 cells at concentrations up to 100 µM for 7 days is accompanied with a significant decrease in the transcript level of CLDN3, CLDN4 and OCLN (Romero et al. 2016). The exact mechanisms underlying the gut barrier dysfunction induced by T-2/HT-2 toxin are unknown and would require further investigations.

### Nivalenol

Nivalenol (NIV) is one of the less studied type B trichothecenes, and little is known about the toxicity of NIV in humans (EFSA 2013). Some studies suggest that exposure to dietary NIV could be associated with an increased incidence of esophageal and gastric cancers in certain regions of China (EFSA 2013; Escriva et al. 2015; Hsia et al. 2004). However, IARC has classified NIV as a group 3 carcinogen (not carcinogenic to humans) due to inadequate evidence for the carcinogenicity in both experimental animals and humans (IARC 1993). NIV is usually found together with DON, and synergistic interactions between them are assumed (Alassane-Kpembi et al. 2013). DON and NIV share highly similar chemical structures, and the only difference between them is a single oxygen atom at the C-4 position in the trichothecene structure (hydrogen and hydroxyl group at the C-4 position in DON and NIV, respectively) (Escriva et al. 2015; Shank et al. 2011; Wu et al. 2013). Although less prevalent in food commodities, NIV is generally believed to have a higher toxicity than DON (Bianco et al. 2012; Cheat et al. 2015; Pinton and Oswald 2014). Unlike DON, NIV inhibits protein synthesis by inhibiting the initiation step of protein translation through interaction with peptidyl transferase at the 60S ribosomal subunit (Rocha et al. 2005). Using different approaches, the effects of NIV on intestinal epithelial cells have been acknowledged. Recently, it has been reported that NIV induces oxidative stress in IEC-6 cells (non-tumorigenic rat intestinal epithelial cell line) by generation of ROS and inducible nitric oxide synthase (iNOS), which leads to activation of nuclear factor kappa B (NF-κB) and nuclear factor erythroid 2-related factor 2 (Nrf2) pathways (Del Regno et al. 2015). A study conducted by Bianco et al. (2012) showed that NIV induces apoptosis in IEC-6 cells by inhibition of the anti-apoptotic protein B cell lymphoma-2 (BCL-2) and the induction of the pro-apoptotic protein Bcl-2-associated X protein (BAX) as well as caspase-3 activation. Induction of apoptosis was further confirmed in ex vivo pig jejunal explant and in vivo pig intestinal loops (Cheat et al. 2015). However, the effect of NIV on the intestinal barrier function has not been studied yet.

### Deoxynivalenol and its mono-acetylated derivatives

Deoxynivalenol (DON) is believed to be the most widely distributed trichothecene (Escriva et al. 2015; Pestka 2010b). The high incidence of human exposure is confirmed by the analysis of urine samples for DON and its glucuronides, demonstrating that the exposure incidence exceeds 90 % of the tested population in many cases (Hepworth et al. 2012; Sarkanj et al. 2013; Turner et al. 2011; Wang et al. 2014; Warth et al. 2012). Human exposure to DON can cover all age groups, even the developing fetus, since DON crosses the placental barrier (Danicke et al. 2007; Nielsen et al. 2011). Genotoxicity and mutagenicity of DON is widely studied, and IARC has classified DON as a group 3 carcinogen (not carcinogenic to humans) (IARC 1993). DON modulates the function of various organ systems. For example, DON is also known as vomitoxin, since it induces a strong emetic effect due to an interaction with the dopaminergic system in the central nervous system (Maresca 2013; Pestka 2010b; Sobrova et al. 2010). Other neurological effects of DON in regulating overall activity and satiety have recently been discussed (Bonnet et al. 2012; Maresca 2013; Yazar and Omurtag 2008). Another important target of DON is the immune system, and DON can induce both immunostimulatory as well as immunosuppressive responses depending on dose, frequency and duration of exposure. As an example, low-dose exposure to DON triggers immune responses, whereas a high dose leads to leukocyte apoptosis and subsequent immunosuppression (Pestka 2007, 2010b). At the cellular level, DON inhibits protein synthesis (at the elongation–termination step of protein translation) through interaction with the peptidyl transferase at the 60S ribosomal subunit (Rocha et al. 2005). The binding of DON to the ribosome rapidly activates MAPK signaling pathways and induces caspase-mediated apoptosis in a process known as the “ribotoxic stress response” (Pestka 2010b; Rocha et al. 2005; Wu et al. 2014a).

In addition to DON itself, two acetylated derivatives (3-acetyl DON, 3-Ac-DON and 15-acetyl DON, 15-Ac-DON) may simultaneously be produced by *Fusarium* species. Due to similarity in the chemical structure, the mode of action of 3-Ac-DON and 15-Ac-DON is generally considered to be the same as DON (Pestka 2010a, b). Recently, the contribution of plant-derived conjugates, such as glucosides of DON, to overall DON exposure is considered as well (Berthiller et al. 2005).

### Effects of DON, 3-Ac-DON and 15-Ac-DON on intestinal barrier function

The contribution of DON to the loss of intestinal barrier function has been extensively examined in different in vitro, ex vivo and in vivo studies (Table 6). Evidence in different human (Caco-2, T84 and HT-29) as well as porcine (IPEC-1 and IPEC-J2) intestinal epithelial cells has shown that DON induces a concentration- and time-dependent drop in TEER values (Akbari et al. 2014; Diesing et al. 2011a; Kasuga et al. 1998; Maresca et al. 2002; Pinton et al. 2012). It could be concluded that IPEC-1 cells are more sensitive to DON compared to Caco-2 cells as indicated by the DON-induced TEER drop (Pinton et al. 2009). This difference may be associated with different origin and type of these cell lines, as Caco-2 cells are human colon adenocarcinoma cells, while IPEC-1 cells are non-transformed and non-carcinoma cells obtained from porcine small intestines (Alassane-Kpembi et al. 2015; Pestka 2007). Recently, we showed that the DON-induced TEER drop considerably depends on the site of application and this response is much more pronounced when DON is applied to the basolateral, rather than the apical surface of Caco-2 cells (Akbari et al. 2014). The same surface-dependent effect is also observed in IPEC-J2 cells (Diesing et al. 2011a). Using horizontal impedance measurements, we and others could show that DON disintegrates a human Caco-2 cell monolayer within the first few hours of exposure in concentrations as low as 1.5 µM (Akbari et al. 2014; Manda et al. 2015). The DON-induced TEER drop in established epithelial cell monolayers is accompanied with a concentration-dependent increase in the flux of paracellular markers such as mannitol, HRP, LY and 4 kDa FITC-dextran (Table 6) (Akbari et al. 2014; De Walle et al. 2010; Ling et al. 2016; Maresca et al. 2008). Goossens et al. (2012) observed that the decrease in TEER is accompanied with an increase in passage of smaller molecules such as the antibiotics doxycycline and paromomycin across IPEC-J2 cells. This is in line with the assumption that an increased flux of paracellular markers is size selective, since our study with two molecular sizes of FITC-dextran (4 and 40 kDa) revealed that DON exposure induces a significant increase in the flux of 4 kDa FITC-dextran in the Caco-2 cells, but not of 40 kDa FITC-dextran (Akbari et al. 2014). A similar concentration-dependent increase in permeability was observed in pig jejunal explants exposed to 20–50 μM DON for up to 2 h (Pinton et al. 2009). Intestinal barrier breakdown was further confirmed in vivo by our previous study showing that a single oral application of DON (25 mg/kg bw) to mice results in significant increase in 4 kDa FITC-dextran permeability (Akbari et al. 2014). Of clinical relevance is the fact that a DON-induced impairment of intestinal integrity may result in the increased transfer of luminal antigens and bacteria. Pinton et al. (2009) described that DON treatment causes a concentration- and time-dependent increase in translocation of pathogenic *E. coli* across IPEC-1 cell monolayers. Other studies pointed out that DON-induced loss of epithelial barrier function, observed by decrease in TEER and increase in paracellular flux, is correlated with the increase in translocation of commensal *E. coli* across Caco-2 cells (Ling et al. 2016; Maresca et al. 2008). DON-enhanced translocation of *S. typhimurium* is reported in both undifferentiated and differentiated IPEC-J2 cells, although undifferentiated cells are found to be more sensitive in comparison with differentiated cells (Vandenbroucke et al. 2011). DON-induced permeability in various in vitro and in vivo models is accompanied with specific alterations in the expression (at transcriptional and protein levels) as well as distribution of different TJs. An up-regulation in mRNA levels of CLDN3, CLDN4, OCLN and ZO-1 was observed in DON-exposed Caco-2 cells (Akbari et al. 2014; De Walle et al. 2010; Osselaere et al. 2013). CLDNs have been reported to be the most susceptible TJs regarding DON exposure to human intestinal epithelial cells (Akbari et al. 2014; Maresca and Fantini 2010; Pinton et al. 2009, 2012). However, in addition to CLDNs, OCLN and ZO-1 have also been shown to be influenced by DON in porcine intestinal epithelial cells (Diesing et al. 2011a, b; Gu et al. 2014; Ling et al. 2016).

Up-regulation of TJ mRNA is often reported as an effect of DON, whereas at the same time, a significant reduction in the protein level of different TJs is observed (Akbari et al. 2014; Bol-Schoenmakers et al. 2016; De Walle et al. 2010; Ling et al. 2016). Therefore, it is assumed that DON primarily targets the TJ proteins and that the RNA up-regulation needs to be considered as a compensatory mechanism (Akbari et al. 2014; Alizadeh et al. 2015; Bol-Schoenmakers et al. 2016; De Walle et al. 2010; Ling et al. 2016; Osselaere et al. 2013). Another explanation could be that in addition to protein synthesis inhibition (which could explain the decrease in protein level of TJs), DON augments and prolongs the usually transient expression of genes either by transcriptional enhancement or transcript stabilization (leading to increased transcriptional rates of TJs), a mechanism described as superinduction (Azcona-Olivera et al. 1995a, b).

*In vivo* exposure to DON-contaminated diet also significantly affects different TJs, and segment-specific effects of DON are reported to occur throughout the intestine. Our previous study, as an example, indicated that up-regulation of the different CLDNs caused by a gavage with DON is most pronounced in the mouse distal small intestine compared to other segments of the intestine (Akbari et al. 2014). Surprisingly, our group found that even after low-level exposure to DON, which has been generally considered as acceptable in animal feeds, substantial changes occur in markers of intestinal integrity. For example, up-regulation of different TJ proteins were observed alongside the intestine, whereas in the jejunum, the mRNA expression of certain TJs (CLDN4, OCLN, ZO-1 and ZO-2) was down-regulated (Alizadeh et al. 2015). Furthermore, Lessard et al. (2015) also observed the down-regulation of CLDN3, CLDN4 and OCLN mRNA levels in the ileum of pigs consuming a DON-contaminated diet, whereas no effect was observed in the jejunum. A study conducted in broiler chickens showed an up-regulation of CLDN1, CLDN5, ZO-1 and ZO-2 mRNA levels in the ileum after dietary DON exposure, while only CLDN5 was affected in the jejunum (Osselaere et al. 2013).

Bol-Schoenmakers et al. (2016) described that single oral exposure of mice to DON (25 mg/kg bw) results in a time-dependent decrease in the CLDN3 protein expression observed in the duodenum. In addition, several studies demonstrated a decrease in protein expression of CLDN4 and OCLN in pig jejunum and ileum after a DON diet (Bracarense et al. 2012; Pinton et al. 2009). In contrast to other studies, Alizadeh et al. (2015) showed that the protein expression of OCLN is significantly increased in duodenum, jejunum and colon of DON-treated pigs compared to control animals, which is probably related to the low-dose exposure to DON in this study. In addition, DON is able to interrupt the distribution pattern of TJs (including CLDNs, OCLN and ZO-1) as demonstrated within different in vitro as well as in vivo models (Akbari et al. 2014; Diesing et al. 2011a, b). Our recent murine study showed that already 6 h after an oral DON gavage (25 mg/kg bw), an irregular distribution of CLDN1, CLDN2 and CLDN3 has been observed in the distal small intestine, whereas in the colon no differences in the TJ distribution pattern were detected (Akbari et al. 2014).

Unlike well-documented effects of DON on gut barrier, knowledge about toxicity of its acetylated derivatives is still limited and only a few studies have addressed intestinal barrier impairment induced by 3-Ac-DON and 15-Ac-DON (Table 6). Kadota et al. (2013) showed that 15-Ac-DON has a higher potency to affect the permeability of Caco-2 cells compared to DON and 3-Ac-DON. The potency of DON and its acetylated derivatives on the barrier function of IPEC-1 cells are ranked as 15-Ac-DON > DON > 3-Ac-DON based on the decrease in TEER values and the increase in the permeability of 4 kDa FITC-dextran. Measuring the protein expression of CLDNs clearly showed that 15-Ac-DON has a more pronounced effect on the expression of CLDN3 and CLDN4 in IPEC-1 cells compared to DON and 3-Ac-DON (Pinton et al. 2012).

As mentioned above, interaction of DON with the peptidyl transferase at the 60S ribosomal subunit has been associated not only with translational arrest and protein synthesis inhibition, but also with an activation of the intracellular protein kinases (particularly MAPKs) and their downstream signaling partners in a process known as the ribotoxic stress response (Pestka 2010a; Pinton et al. 2012; Plotnikov et al. 2011; Wang et al. 2014). MAPKs play a crucial role in many physiological processes including cell growth, differentiation, apoptosis and immune responses (Plotnikov et al. 2011). Further studies have shown that TJ structure and function can also be regulated by signaling molecules involved in MAPK pathways (Matter and Balda 2003; McLaughlin et al. 2009). At the molecular level, MAPK extracellular signal-regulated kinase 1 and 2 (ERK1/2), c-Jun N-terminal kinase (JNK) and p38 are described to get rapidly activated by DON in human as well as porcine intestinal cell lines (Pinton et al. 2012; Sergent et al. 2006), and this activation leads to a decrease in the expression of CLDNs (Pinton et al. 2010, 2012).

Different observations of DON-induced activation of MAPKs have been reported in ex vivo as well as in vivo models. Using IPEC-1 cells, Pinton et al. (2010) showed that the DON-activated MAPK ERK1/2 correlates with a reduction in barrier function observed by decrease in TEER, increase in paracellular permeability and decrease in the expression of CLDN4. Interestingly, inhibition of ERK1/2 phosphorylation restored the barrier function of differentiated IPEC-1 cells (Pinton et al. 2010). In addition, a study conducted by the same author showed that none of the MAPKs, such as ERK1/2, JNK and p38, are significantly activated neither in ex vivo (pig jejunal explants exposed to DON) nor in in vivo (jejunum of DON-fed pigs) models (Pinton et al. 2012). However, another study using the same ex vivo and in vivo approaches reported that DON significantly enhances the phosphorylation of ERK1/2 and p38, whereas the phosphorylation of JNK remains unaffected (Lucioli et al. 2013).

Only a few studies displayed differences between DON and its acetylated derivatives regarding their potency to activate MAPKs. 15-Ac-DON, as an example, has a greater capacity to activate MAPK ERK1/2, p38 and JNK, in the porcine intestinal epithelial cells and in pig jejunal explants compared to DON and 3-Ac-DON (Pinton et al. 2012).

## Clinical relevance and conclusions

A dynamic and well-regulated intestinal barrier is essential to protect the body against dietary antigens and residential intestinal microbiota. This barrier is created by an impermeable layer of epithelial cells, sealed by specific TJ proteins preventing the paracellular diffusion of luminal antigens and microorganisms (Fig. 1). An impaired intestinal barrier leads to mucosal inflammation and has been linked to the pathogenesis of various chronic intestinal inflammatory diseases, such as Crohn’s disease, ulcerative colitis, celiac disease and irritable bowel syndrome (Bertiaux-Vandaele et al. 2011; Drago et al. 2006; Gibson 2004; Hering et al. 2012; Suzuki 2013; Vetrano et al. 2008). TJ proteins, which seal the epithelial monolayer, are one of the most important functional elements of the intestinal barrier, and a decrease in the abundance and a the re-distribution of different TJ proteins are observed in all major chronic intestinal inflammatory diseases as summarized in Table 7.

**Table 7:** Aspect of TJ-related barrier dysfunction in chronic intestinal inflammatory diseases

Inflammatory disease	TJ proteins	References
Crohn’s disease	↓OCLN, ↓CLDN3, ↓CLDN5, ↓CLDN8, ↓JAM ↑CLDN2 Redistribution of OCLN, CLDN3, CLDN5, CLDN8	Prasad et al. (2005), Vetrano et al. (2008), Zeissig et al. (2007)
Ulcerative colitis	↓OCLN, ↓CLDN1, ↓CLDN4, ↓JAM, ↓Tricellulin ↑CLDN2 Redistribution of OCLN, CLDN1, CLDN4	Heller et al. (2005), Hering et al. (2012), Prasad et al. (2005), Vetrano et al. (2008)
Celiac disease	↓OCLN, ↓ZO-1 ↑CLDN2, ↑CLDN3 Redistribution of OCLN	Drago et al. (2006), Szakal et al. (2010)
Irritable bowel syndrome	↓OCLN, ↓CLDN1, ↓ZO-1 Redistribution of OCLN, CLDN1, ZO-1	Bertiaux-Vandaele et al. (2011)

Expression: ↓decrease, ↑increase

Dietary exposure of humans and animals to mycotoxins is of growing concern due to the apparently still increasing prevalence of these fungal toxins in food and feed commodities (Bhat et al. 2010; Marin et al. 2013; Rodrigues and Naehrer 2012; Wu et al. 2014a). Due to this increasing prevalence in food commodities, mycotoxins appear to be important, but often neglected substances that are able to affect TJ proteins and impair the integrity of the intestinal barrier. Even though mycotoxins have not been associated with a specific intestinal disease, the investigations summarized above demonstrate that mycotoxins impair the expression and function of TJ proteins in different ways. Among the various mycotoxins, particularly DON has been identified to modulate the expression, intracellular localization and function of TJ proteins (Fig. 1), while PAT seems to directly affect the epithelial cell monolayer. PAT is only found incidentally as a contaminant of fruit juices and other fruit products, whereas DON is found in major food supplies, such as wheat and other cereal products, which are consumed daily. This continuing exposure suggests a role of this mycotoxin in the etiology of chronic intestinal inflammatory diseases. The observation that even pathogenic bacteria are translocated from the intestinal lumen to the internal environment, when animals are challenged with mycotoxins confirms their significance in inflammatory reactions. Moreover, considering the apparent lactational transfer of various mycotoxins (transfer from maternal plasma into milk), exposure of infants deserves special attention. Even minor changes in the (developing) barrier function can lead to exposure to luminal antigens in early phases of life and may result in accelerated immunological responses and clinical manifestations, such as allergies in later stages of life. The prevalence of wheat allergy in children is increasing (Cianferoni et al. 2013; Makela et al. 2014; Sievers et al. 2015), and as DON is mainly found in wheat and wheat-derived products, it cannot be excluded that DON plays also a role in the onset of allergic reactions in children. Further studies should be devoted to the effects of frequently occurring mycotoxins in human food supplies on TJ proteins, and their effect on the intestinal barrier should be included in the overall risk assessment of mycotoxins in foods.

## Electronic supplementary material

Below is the link to the electronic supplementary material.

Supplementary material 1 (DOCX 64 kb)

## Abbreviations

3-Ac-DON: 3-Acetyl deoxynivalenol
15-Ac-DON: 15-Acetyl deoxynivalenol
AFB_1_: Aflatoxin B_1_
AFM_1_: Aflatoxin M_1_
α-ZOL: Alpha-zearalenol
β-ZOL: Beta-zearalenol
BEN: Balkan endemic nephropathy
CLDNs: Claudins
CYP: Cytochrome P450
DON: Deoxynivalenol
ERK1/2: Extracellular signal-regulated kinase 1 and 2
FB_1_: Fumonisin B_1_
FITC: Fluorescein isothiocyanate
GI tract: Gastrointestinal tract
GSH: Glutathione
HRP: Horseradish peroxidase
IEC: Intestinal epithelial cell
IARC: International agency for research on cancer
iNOS: Inducible nitric oxide synthase
JAM: Junctional adhesion molecule
JNK: c-Jun N-terminal kinase
LY: Lucifer yellow
MAPKs: Mitogen-activated protein kinases
NIV: Nivalenol
OCLN: Occludin
OTA: Ochratoxin A
PAT: Patulin
ROS: Reactive oxygen species
RNS: Reactive nitrogen species
TEER: Transepithelial electrical resistance
TJs: Tight junction proteins
ZEA: Zearalenone
ZO: Zonula occludens
