# Supplementary material for: The intestinal barrier as an emerging target in the toxicological assessment of mycotoxins
Source: Arch Toxicol. 2016 Jul 14;91(3):1007–29. doi: 10.1007/s00204-016-1794-8 (PMC5316402; doi:10.1007/s00204-016-1794-8)
Supplement: Supplementary file 1 — Supplementary material 1 (DOCX 64 kb) [file 204_2016_1794_MOESM1_ESM.docx]

**Electronic Supplementary Material**

The rapid appearance of most mycotoxins in the blood circulation clearly indicates that the majority of the ingested toxin is absorbed. Using Caco-2 cells, it has been shown that mycotoxins may enter the cell by passive diffusion, whereas efflux of mycotoxins across the epithelial membranes (either apically or basolaterally) is mediated by membrane transport proteins, in particular ATP-binding cassette transporters (ABC transporters) ([Berger et al. 2003](#_ENREF_1); [Caloni et al. 2005, 2006, 2012](#_ENREF_2); [De Angelis et al. 2005](#_ENREF_5); [Gratz et al. 2007](#_ENREF_6); [Kadota et al. 2013](#_ENREF_7); [Pfeiffer et al. 2011](#_ENREF_8); [Schrickx et al. 2006](#_ENREF_10); [Sergent et al. 2005](#_ENREF_11); [Tep et al. 2007](#_ENREF_13); [Videmann et al. 2007](#_ENREF_14), 2009). Caco-2 cells are known to express the ABC transporters: P-glycoprotein (P-gp, ABCB1), multidrug resistance-associated proteins 1-6 (MRP1-MRP6, ABCC1-6) and breast cancer-resistant protein (BCRP, ABCG2) ([Prime-Chapman et al. 2004](#_ENREF_9); [Taipalensuu et al. 2001](#_ENREF_12); [Xia et al. 2005](#_ENREF_16)). The ABC transporters involved in the efflux of mycotoxins in Caco-2 cells are summarized in Table 1.

ESM-Table 1 Permeability of the Caco-2 cell monolayer for individual mycotoxins

| **Mycotoxin** | **Potential ABC transporters** | **References** |
| --- | --- | --- |
| AFB_1_, AFM_1_ | ND |  |
| ZEA,  α-ZOL,  β-ZOL | ZEA: MRP1, MRP2  α-ZOL: MRP1, MRP2  β-ZOL: MRP2, MRP3 | ([Videmann et al. 2009](#_ENREF_14)) |
| OTA | MRP2, BCRP | ([Berger et al. 2003](#_ENREF_1))  ([Schrickx et al. 2006](#_ENREF_10)) |
| Patulin | ND |  |
| FB_1_ | P-gp | ([Caloni et al. 2005](#_ENREF_4))  ([De Angelis et al. 2005](#_ENREF_5)) |
| T-2 toxin | ND |  |
| NIV | P-gp, MRP2 | ([Tep et al. 2007](#_ENREF_13)) |
| DON, 3-Ac-DON, 15-Ac-DON | P-gp, MRP2 | ([Videmann et al. 2007](#_ENREF_15))  ([Kadota et al. 2013](#_ENREF_7)) |

Abbreviations used: 3-Ac-DON, 3-acetyl deoxynivalenol; 15-Ac-DON, 15-acetyl deoxynivalenol; AFB_1_, aflatoxin B_1_; AFM_1_, aflatoxin M_1_; α-ZOL, alpha-zearalenol; β-ZOL, beta-zearalenol; DON, deoxynivaleol; FB_1_, fumonisin B_1_; ND, not determined; NIV, nivalenol; OTA, ochratoxin A; ZEA, zearalenone.

**References**

Berger V, Gabriel AF, Sergent T, Trouet A, Larondelle Y, Schneider YJ (2003) Interaction of ochratoxin A with human intestinal Caco-2 cells: possible implication of a multidrug resistance-associated protein (MRP2). Toxicol Lett 140-141:465-476. doi:10.1016/S0378-4274(03)00043-2

Caloni F, Cortinovis C, Pizzo F, De Angelis I (2012) Transport of Aflatoxin M(1) in Human Intestinal Caco-2/TC7 Cells. Front Pharmacol 3:111. doi:10.3389/fphar.2012.00111

Caloni F, Stammati A, Frigge G, De Angelis I (2006) Aflatoxin M1 absorption and cytotoxicity on human intestinal in vitro model. Toxicon 47:409-415. doi:10.1016/j.toxicon.2005.12.003

Caloni F, Stammati AL, Raimondi F, De Angelis I (2005) In vitro study with Caco-2 cells on fumonisin B1: aminopentol intestinal passage and role of P-glycoprotein. Vet Res Commun 29 Suppl 2:285-287. doi:10.1007/s11259-005-0063-8

De Angelis I, Frigge G, Raimondi F, Stammati A, Zucco F, Caloni F (2005) Absorption of fumonisin B1 and aminopentol on an in vitro model of intestinal epithelium; the role of P-glycoprotein. Toxicon 45:285-291. doi:10.1016/j.toxicon.2004.10.015

Gratz S, Wu QK, El-Nezami H, Juvonen RO, Mykkanen H, Turner PC (2007) Lactobacillus rhamnosus strain GG reduces aflatoxin B1 transport, metabolism, and toxicity in Caco-2 Cells. Appl Environ Microbiol 73:3958-3964. doi:10.1128/AEM.02944-06

Kadota T, Furusawa H, Hirano S, Tajima O, Kamata Y, Sugita-Konishi Y (2013) Comparative study of deoxynivalenol, 3-acetyldeoxynivalenol, and 15-acetyldeoxynivalenol on intestinal transport and IL-8 secretion in the human cell line Caco-2. Toxicol In Vitro 27:1888-1895. doi:10.1016/j.tiv.2013.06.003

Pfeiffer E, Kommer A, Dempe JS, Hildebrand AA, Metzler M (2011) Absorption and metabolism of the mycotoxin zearalenone and the growth promotor zeranol in Caco-2 cells in vitro. Mol Nutr Food Res 55:560-567. doi:10.1002/mnfr.201000381

Prime-Chapman HM, Fearn RA, Cooper AE, Moore V, Hirst BH (2004) Differential multidrug resistance-associated protein 1 through 6 isoform expression and function in human intestinal epithelial Caco-2 cells. J Pharmacol Exp Ther 311:476-484. doi:10.1124/jpet.104.068775

Schrickx J, Lektarau Y, Fink-Gremmels J (2006) Ochratoxin A secretion by ATP-dependent membrane transporters in Caco-2 cells. Arch Toxicol 80:243-249. doi:10.1007/s00204-005-0041-5

Sergent T, Garsou S, Schaut A, De Saeger S, Pussemier L, Van Peteghem C, Larondelle Y, Schneider YJ (2005) Differential modulation of ochratoxin A absorption across Caco-2 cells by dietary polyphenols, used at realistic intestinal concentrations. Toxicol Lett 159:60-70. doi:10.1016/j.toxlet.2005.04.013

Taipalensuu J, Tornblom H, Lindberg G, Einarsson C, Sjoqvist F, Melhus H, Garberg P, Sjostrom B, Lundgren B, Artursson P (2001) Correlation of gene expression of ten drug efflux proteins of the ATP-binding cassette transporter family in normal human jejunum and in human intestinal epithelial Caco-2 cell monolayers. J Pharmacol Exp Ther 299:164-170.

Tep J, Videmann B, Mazallon M, Balleydier S, Cavret S, Lecoeur S (2007) Transepithelial transport of fusariotoxin nivalenol: mediation of secretion by ABC transporters. Toxicol Lett 170:248-258. doi:10.1016/j.toxlet.2007.03.012

Videmann B, Mazallon M, Prouillac C, Delaforge M, Lecoeur S (2009) ABCC1, ABCC2 and ABCC3 are implicated in the transepithelial transport of the myco-estrogen zearalenone and its major metabolites. Toxicol Lett 190:215-223. doi:10.1016/j.toxlet.2009.07.021

Videmann B, Tep J, Cavret S, Lecoeur S (2007) Epithelial transport of deoxynivalenol: involvement of human P-glycoprotein (ABCB1) and multidrug resistance-associated protein 2 (ABCC2). Food Chem Toxicol 45:1938-1947. doi:10.1016/j.fct.2007.04.011

Xia CQ, Liu N, Yang D, Miwa G, Gan LS (2005) Expression, localization, and functional characteristics of breast cancer resistance protein in Caco-2 cells. Drug Metab Dispos 33:637-643. doi:10.1124/dmd.104.003442
